# Determination of coefficient defining leaf area development in different genotypes, plant types and planting densities in peanut (*Arachis hypogeae* L.)

**DOI:** 10.1016/j.fcr.2016.09.013

**Published:** 2016-12

**Authors:** Oumarou Halilou, Halime Mahamat Hissene, José A. Clavijo Michelangeli, Falalou Hamidou, Thomas R. Sinclair, Afshin Soltani, Saadou Mahamane, Vincent Vadez

**Affiliations:** a International Crops Research Institute for the Semi-Arid Tropics (ICRISAT), Sahelian Center, Niamey, Niger; b Department of Biology, Faculty of Sciences, University Abdou Moumouni, Niamey, Niger; c Centre d’Etude Régional pour l’Amélioration de l’Adaptation à la Sécheresse, Thiès-Escale, Senegal; d Department of Crop Sciences, North Carolina State University, Raleigh, NC 27695-7620, USA; e Agronomy Group, Gorgan University of Agricultural Sciences and Natural Resources, Gorgan 49138-15739, Iran; f International Crops Research Institute for the Semi-Arid Tropics (ICRISAT), Patancheru, Greater Hyderabad 502324, Telangana, India

**Keywords:** Phyllochron, Leaf area, Peanut, Allometric model, Degree-days

## Abstract

Rapid leaf area development may be attractive under a number of cropping conditions to enhance the vigor of crop establishment and allow rapid canopy closure for maximizing light interception and shading of weed competitors. This study was undertaken to determine (1) if parameters describing leaf area development varied among ten peanut (*Arachis hypogeae* L.) genotypes grown in field and pot experiments, (2) if these parameters were affected by the planting density, and (3) if these parameters varied between Spanish and Virginia genotypes. Leaf area development was described by two steps: prediction of main stem number of nodes based on phyllochron development and plant leaf area dependent based on main stem node number. There was no genetic variation in the phyllochron measured in the field. However, the phyllochron was much longer for plants grown in pots as compared to the field-grown plants. These results indicated a negative aspect of growing peanut plants in the pots used in this experiment. In contrast to phyllochron, there was no difference in the relationship between plant leaf area and main stem node number between the pot and field experiments. However, there was genetic variation in both the pot and field experiments in the exponential coefficient (PLAPOW) of the power function used to describe leaf area development from node number. This genetic variation was confirmed in another experiment with a larger number of genotypes, although possible G × E interaction for the PLAPOW was found. Sowing density did not affect the power function relating leaf area to main stem node number. There was also no difference in the power function coefficient between Spanish and Virginia genotypes. SSM (Simple Simulation model) reliably predicted leaf canopy development in groundnut. Indeed the leaf area showed a close agreement between predicted and observed values up to 60000 cm^2^ m^−2^. The slightly higher prediction in India and slightly lower prediction in Niger reflected GxE interactions. Until more understanding is obtained on the possible GxE interaction effects on the canopy development, a generic PLAPOW value of 2.71, no correction for sowing density, and a phyllochron on 53 °C could be used to model canopy development in peanut.

## Highlights

• A vigorous canopy development can shade weed competitors and enhance light interception.
• We assess whether the coefficients that characterize the canopy development in a peanut crop model vary with genotype, density, and botanical type.
• There was variation among genotype for the leaf canopy development parameters.
• Botanical type and density did not alter the canopy development parameters.
• The model predicted the leaf canopy development well.

## 1 Introduction

Crops produce leaves to intercept light, use intercepted light energy to synthesize mass, and partition mass into grain. Rapid establishment of leaf area also allows for diminished water evaporation from the soil surface and for shading of an emerging weed. To understand leaf area development, allometric relationships have been developed in many crops between leaf node number and plant leaf area during the major phase of leaf area development (Sinclair, 1984, Robertson et al., 2002, Soltani et al., 2006). In crop simulations, total plant leaf area (PLA) is then calculated as an empirical function of main stem node number. The crop model APSIM first estimates the effective leaf number for the entire plant based on main stem node number (Hammer et al., 1995, Robertson et al., 2002). The specific steps in this approach are calculation of: (1) node number on main-stem based on cumulative temperature, (2) total plant leaf number from main stem node number, (3) fraction of senesced leaf number on main stem based on cumulative temperature, (4) plant senesced leaf number from main stem senesced leaf number, (5) green leaf number from total and senesced leaves, (6) individual leaf size from main stem node number or cumulative temperature (assumed to be constant 40 cm^2^ in peanut), (7) PLA as the product of total leaf number per plant and individual leaf size, (8) leaf area index (LAI) from plant leaf area and plant density. Clearly, this method requires several functions and a number of defined coefficients. An alternative proposed by Soltani and Sinclair (2012) in the model SSM (Simple Simulation Model) is to avoid the assumption about individual leaf area by simply calculating plant leaf area directly from an empirical function based on main stem node number. Therefore, this simpler approach requires only three steps by calculating: (1) main stem node number from cumulative temperature, (2) PLA from main stem node number [in this step density effect is considered], (3) LAI from PLA and plant density. This simplified approach requires fewer parameters than the APSIM method, which may allow easier experimental evaluation of parameters. In addition, it includes a specific target parameter for considering a plant density effect. It is this simpler model we use here to evaluate PLA development in several peanut genotypes.

Under non water stressed conditions, the rate of node number appearance on the main stem is based on daily temperature units, which is commonly calculated as the difference between daily mean temperature and a base temperature. The cumulative temperature units (cumulated °C or °C) required for the production of successive nodes on the main stem is defined as the phyllochron. In peanut, node production on the main stem starts after plant emergence and continues up to final harvest with slower development after the appearance of the 17th node (Forestier, 1969). Leong and Ong (1983) found a decrease in the rate of node number appearance, or leaf appearance rate, under drought conditions with a base temperature up to 11.4 °C. Young et al. (1979) reported for peanut (*Arachis hypogaea* L.) that the base temperature for leaf appearance among several genotypes was 10° C, meaning that there was no leaf development below this temperature. Peanut base temperature can also vary with botanical type. Bagnall and King (1991) identified different phenological base temperatures for Spanish genotypes (13.6 °C), Valencia genotypes (12.6 °C) and Virginia genotypes (11.4 °C). Mohamed (1984) found that base temperature for peanut ranged from 8 to 11.5 °C in experiments with maximum temperature ranging from 29 to 36.5 °C. Most of peanut models of leaf development (Fortanier, 1957, Ong, 1986, Boote et al., 1989) use 11 °C as a generic base temperature, and this is the base temperature used by the model in this work. The daily temperature units increase linearly above the base temperature up to the optimum temperature, which is often assumed to be 28 °C in peanut.

A key question in crop improvement is whether genetic variability exists in the parameters describing leaf area increase. That is, is there genetic variability that might be exploited to breed for altered rate of leaf area development? For example, in environments with available water it could be advantageous to have rapid leaf area development to allow early crop vigor and to shade weed competitors. In studies of phyllochron diversity, Dofing (1999) found in barley (*Hordeum vulgare* L.) a range among genotypes of 52 to 70 °C and Rebolledo et al. (2012) found in rice (*Oryza sativa* L.) a range of about 45 to 71 °C. On the other hand, van Esbroeck et al. (2008) reported little variation in phyllochron across a diverse panel of maize genotypes. The same observation was found in some cereals where phyllochron was found to be almost constant from seedling stage to flag-leaf expansion in sorghum (*Sorghum bicolor* L.) (Muchow and Carberry, 1990, Craufurd et al., 1998, Clerget et al., 2008), millet (*Pennisetum glaucum* L.) (Craufurd and Bidinger, 1988), maize (*Zea mays* L.) (Birch et al., 1998). Sinclair (1984) also found that the phyllochron was constant with variation of base temperature among soybean (*Glycine max* (L.) Merr.) genotypes. Leong and Ong (1983) and Craufurd et al. (1997) reported that the phyllochron was stable across environments for single genotypes of peanut (56 °C). There appears to be no information on the genetic variation in peanut for phyllochron and the calculation of plant leaf area. Therefore, a major objective to this investigation was to document the variation in leaf area parameters across ten peanut genotypes of diverse genetic background, including a comparison of Spanish and Virginia types.

Another key question was whether plant density affects leaf area development in peanut, and can this be described in the proposed parameters describing leaf area development. In peanut, a recommended seeding rate of 60 kg ha^−1^ is common in Africa and this leads to a density of approximately 15–20 plant m^−2^. In India higher sowing density is used resulting in greater than 30 plant m^−2^ and in Australia 6.5 to 7.5 plants m^−2^ (Virginia-type) and >22.5 plants m^−2^ for (Spanish-type) (Bell et al., 1991). It was previously reported (Giayetto et al., 1998) in peanut (runner and erect types) that increased plant density resulted in a decrease in individual PLA and plant dry matter. These decreases were attributed to greater intraspecific competition produced by the shortening of distances between rows. A study in four Virginia type cultivars showed that an increased plant density led to increased vegetative development and more numerous reproductive organs, although this did not led to higher yield because of indeterminacy in pod setting (Cahaner and Ashri, 1974). On the contrary a study in two Virginia type cultivars showed no increase in the vegetative growth and yield at higher density (Tewolde et al., 2002). In any case, none of these studies generated the data necessary to quantitatively evaluate the parameters in the functions proposed to describe PLA or a range of peanut genotypes and plant densities.

Therefore, the objectives of this work were to quantify PLA development in peanut and use the derived parameters to assess possible genetic variation and density effects. The specific basis of comparison was: (i) phyllochron and the coefficient relating node number on the main stem and leaf area; (ii) effect of sowing density on these parameters. A side objective was to compare the generation of these coefficients in the field and in small pots.

## 2 Material and methods

### 2.1 Plant material

**Fig. 1:**
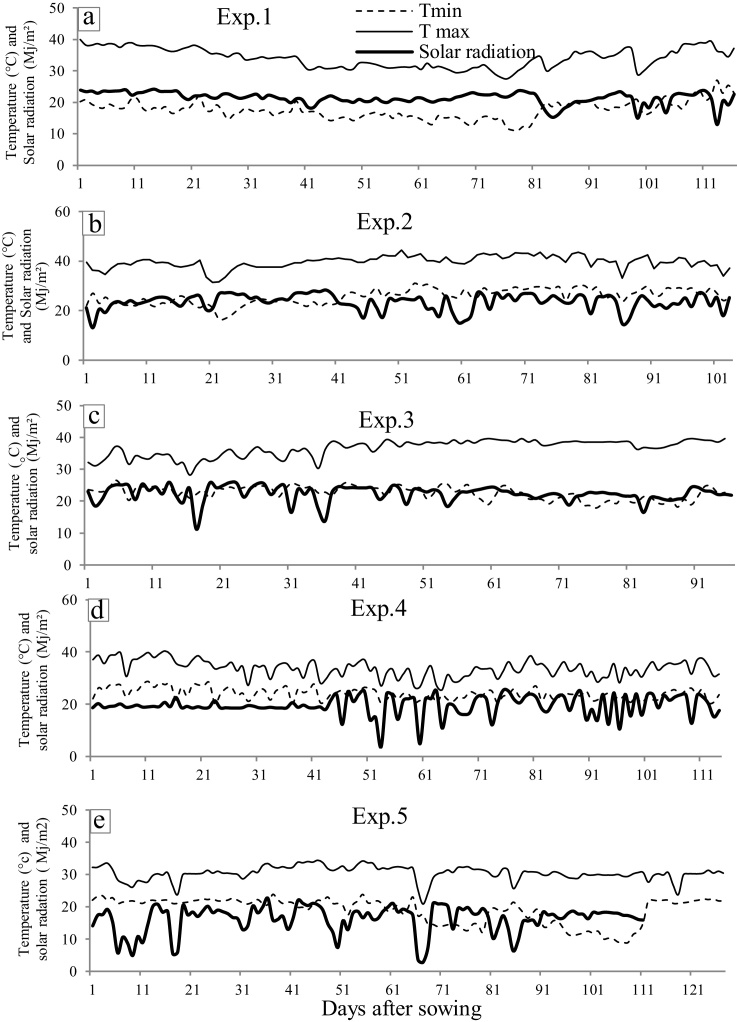
Climatic data (Temperature max and min, Solar radiation – see legend in top pane ‘a’) during five experiments (a,b,c,d,e corresponding to experiment 1,2,3,4,5 respectively). T_min_ = minimal temperature (°C), T_max_ = maximal temperature (°C), solar radiation MJ/m^2^.

One pot experiment and four field experiments were conducted. The pot experiment (Exp. 1) was carried out at the end of the rainy season (October 2011 to January 2012, Fig. 1a) at the ICRISAT Sahelian Centre in (Sadoré, Niger, 45 km south of Niamey city, 13°N, 2°E). Three field experiments were also conducted at this location. Exp. 2 was done during the summer season (February to May 2012, Fig. 1b). Exp 3, was performed during and after the end of the rainy season 2012 (September to December, Fig. 1.c). Exp 4 was done during the rainy season 2014 (June to September, Fig. 1d). In addition, a field experiment (Exp 5) was done during the rainy season 2014 (August-December 2014 Fig. 1e) at the ICRISAT headquarter (Patancheru, India). The same ten peanut genotypes (55–437, ICGV 00350, ICG 12697, FLEUR 11, ICG 4750, TMV2, JL24, ICGV 91114, ICG 3584, ICG 1834), all Spanish botanical types, were included in Exp.1 and Exp.2. These genotypes were selected from the ICRISAT reference collection because of indications of contrasting differences in leaf area development under field tests in India and Niger. In Exp.3 aiming at exploring a larger range of genotypes for possible genotypic differences in the parameter controlling the leaf canopy development, a set of 20 Spanish genotypes was used (ICG 97183, ICGV 97182, ICGV 02266, ICGV 02189, ICG 11088, ICG 8751, ICGV 01232, ICGS 44, ICG 15287, ICGV 99001, in addition to those of Exp.1). In Exp.4, two Spanish genotypes (Fleur11 and ICG1834) and two Virginia genotypes (ICG13723 and ICG2777) were used. In Exp.5, two Spanish genotypes (Fleur11 and ICG1834) and two Virginia genotypes (ICG4598 and ICG2777) were used.

### 2.2 Pot experiment

Plants were grown outdoors in plastic pots (6 L, 25-cm diameter × 20-cm height). The pots were placed on a plastic transparent sheet on the soil surface. Each pot was filled with 8 kg of mixed soil in proportion of 56% sand; 33% clay and 11% farm yard manure. In each pot, 2 g of carbofuran was incorporated into the top 3-cm surface soil to avoid damage by soil-borne pests, and 2 g of di-ammonium phosphate for initial fertilization. The day before sowing, pots were watered to field capacity. Three seeds treated with fungicide (Thyoral) were sown in each pot and 12 d after sowing; each pot was thinned to one plant per pot. Pots were kept under well-watered condition during the entire duration of the experiment.

The experimental design was a randomized complete block design with 10 blocks each with 5 replications of each of the 10 genotypes. Therefore, a total of 50 pots were sown for each genotype. Leaf appearance on the main stem was recorded each day, on five plants per genotype in one fixed block through the growing season to final harvest. From 15 DAS up to final harvest, a block was destructively harvested every 10 d for measurement of leaf area. Plant leaf area for each plant was measured with an area meter (LI-3100, Li-Cor, inc, Lincoln, Nebraska, USA).

### 2.3 Field experiments

The soils at the ICRISAT Sahelian Centre are arenosols (World Reference Base) with very low water holding capacity, low pH, low inherent soil fertility and organic matter content.

Before sowing, 15–15–15 (N-P_2_O_5_-K_2_O) fertilizer at 200 kg ha^−1^, and farmyard manure (2000 kg ha^−1^) were incorporated into the soil. In Exp 2, the field was plowed and irrigated twice one day before sowing to ensure the profile was fully moisted. Seeds were sown by hand; the 10 entries were sown in three replicated plots arranged in a randomized complete block design. Each plot (2 m^2^) contained two rows (2-m long, 50-cm distance between rows), with a spacing of 10 cm between plants within a row, giving a total of 40 plants per plot. Plants were irrigated two times per week with 25 mm of water each time, using a linear movement system (Valmont Irrigation Inc., Valley, Nebraska, USA). Calcium-ammonium-nitrate (200 kg ha^−1^) and gypsum (200 kg ha^−1^) were applied one week after 50% flowering. Daily observations were done on two tagged plants in each plot in three replications to record new leaf appearance on the main stem. Leaf appearance date was recorded for each leaf when the leaf was fully expanded. Sixteen days after sowing, plants from an area of 0.5 m^2^ were harvested from each plot for measurement of leaf area. This process was repeated at 40 and 70 DAS when three plants per plot were harvested. The leaf area of each plant at each harvest was measured separately with an area meter (LI-3100, Li-Cor, inc, Lincoln, Nebraska, USA).

Exp 3 was carried out following the same procedure as Exp 2, the only difference being the larger number of genotypes. The samplings were done at 21, 42 and 60 DAS.

In addition, two experiments (Exp 4 and 5) were undertaken to assess possible effects of sowing density on the leaf canopy development parameters, one in India and one in Niger. In Niger the density experiment was conducted in ICRISAT Sadoré (see Exp.2 details). The maximum/minimum temperature and relative humidity was respectively 40.3/20.2 °C and 98/34% RH during the experiment (Fig. 1d). Four genotypes, two Spanish (FLEUR11 and ICG1834) and two Virginia (ICG13723 and ICG2777) genotypes were sown in three replicated plots, with four densities (10, 20, 30, 40 plant m^−2^) arranged in a randomized complete block design. In the lowest densities (10 and 20 plant m^−2^), each plot (4 m^2^) contained two rows (4-m long, 50-cm distance between rows). The 10 plant m^−2^ density was achieved by 20 cm spacing between plants within a row, while the 20 plant m^−2^ density was obtained by 10 cm spacing between plants within a row. In densities 30 and 40 plant m^−2^, each plot (4 m^2^) contained four rows (4 m long, 25 cm distance between rows). For density 30 plant m^−2^, spacing between plants within a row of 14–15 cm was used. For 40 plant m^−2^, a spacing of 10 cm between plants within a row was used. A total of six samplings were done at 15, 25, 40, 55, 70 and 85 day after sowing (DAS). At each sampling, 5 plants from an area of 0.3 m^2^ were harvested from each plot for measurement of leaf area and node number. The leaf area of each plant at each harvest was measured separately with an area meter (LI-3100, Li-Cor, inc, Lincoln, Nebraska, USA).

In India, the density experiment was conducted at ICRISAT Patancheru (17.53°N, 78.27°E, Altitude: 545m). The maximum/minimum temperature and relative humidity was respectively 34.4/8.9 °C and 98/25% RH during the experiment (Fig. 1e). Sowing was done on 22/08/2014, on red sandy soil (Alfisol). Soil was fertilized with Single Super Phosphate (SSP, 275 kg/ha) before sowing. During the whole experimental period, the field was fully controlled against insects attack. The field was irrigated regularly once every one or two weeks using drip irrigation (at a rate of 10 mm h^−1^ for four to five hours). Four genotypes (two Spanish genotypes (Fleur11 and ICG1834) and two Virginia genotypes (ICG4598 and ICG2777)) were sown in three replicated plots, with three densities (25, 33 and 50 plant m^−2^), row-to-row distance was 33 cm and plants were spaced at 12m, 9 cm, and 6 cm respectively. A total of four samplings (32, 46, 59, 74 DAS) were done. At the first sampling (32 DAS), 40 cm of 4 rows was harvested and for the three others dates (46, 59, 74 DAS) 30 cm of 4 rows was harvested. The nod number and the leaf area (LI-3100, Li-Cor, inc, Lincoln, Nebraska, USA) were estimated on these samples to evaluate the Plapow (parameter relating node number on the main stem and leaf area). The leaf area of each plant at each harvest was measured separately with an area meter (LI-3100, Li-Cor, inc, Lincoln, Nebraska, USA). The phenological parameters (flowering, pod appearance and maturity period) were recorded.

### 2.4 Leaf area development model

Plant leaf area calculation in the model was done in two stages as described by Soltani and Sinclair (2012). First, main stem node number is predicted as a function of temperature. Then, the PLA is computed based on main stem node number (Soltani et al., 2006).

The prediction of daily increase in main stem node number was calculated based on the temperature to which the plants were exposed on each day, and therefore on the accumulated daily thermal time. A 3-piece segmented function of temperature, (f(T)) (Soltani et al., 2006) was used to make these calculations. This function computes a multiplication factor, which varies between 0 and 1, that is used for the calculation of daily thermal time, such that:

(1) f (T) = (T − T_b_)/(T_o1_ − T_b_) if T_b_ < T < T_o1_

f (T) = (T_c_ − T)/(T_c_−T_o2_) if T_o2_ < T < T_c_

f (T) = 1 if T_o1_ ≤ T ≤ T_o2_

f (T) = 0 if T ≤ T_b_ or T ≥ T_c_

Where T_b_ is the base temperature, T_o1_ is the lower optimum temperature, T_o2_ is the upper optimum temperature and T_c_ is the ceiling temperature. The cardinal temperature for the base temperature was assumed to be 11 °C and the lower optimum was 28 °C as discussed in the Introduction. The upper optimum was assumed to be 32 °C and the ceiling temperature was assumed to be 55 °C (Boote et al., 1989). The daily thermal time (DTT) accumulation is calculated as:

DTT = (T_o1_ − T_b_) * f(T)

Then the daily increase in main stem node number is calculated as DTT/phyllochron. For each genotype, the main stem node number was plotted against cumulative temperature units (Ranganathan et al., 2001). The slope of this plot yielded the leaf appearance rate (leaf per °C), i.e. the inverse of the phyllochron (°C).

Potential plant leaf area (PLA, cm^2^) is computed from the main stem node number (MSNN) using a one-parameter, power function (Soltani et al., 2006),

(2) PLA = MSNN^PLAPOW^

Where PLAPOW is the exponential coefficient of the power relationship between plant leaf area and node number on the main stem.

### 2.5 Model robustness in predicting leaf area development

Exp. 4 and 5 were also used to assess the leaf area of the canopy at different stages in the course of the crop development. Six samplings (15, 25, 40, 55, 70, 85 DAS) were done in the Niger experiment, and four samplings (32, 46, 59, 74 DAS) in the India experiment.

### 2.6 Statistical analysis

The results of phyllochron and leaf area were analyzed using respectively, GENSTAT program version 10 (Genstat, Release 10.1) and SAS system, NLIN Procedure with Gauss-Newton Method. The analysis of variance was done with the general ANOVA. The range of variation of the parameterin the relationship between plant leaf area and node number on the main stem was analyzed using the nonlinear regression with the power function. This analysis provided a standard error and a 95% confidence interval for each threshold value for each genotype. The analysis was done using GraphPad Prism (GraphPad Prism 5, San Diego, CA, USA).

## 3 Results

### 3.1 Phyllochron

Peanut node numbers increased linearly essentially over the entire range of accumulated temperature units in both pot and field experiments (Table 1). The R^2^ for the regressions of the pot results were 0.86 or greater and for the field results were 0.94 or greater. The phyllochron obtained from the regression analysis for the pot experiment varied among genotypes from 81 to 102 °C with a mean of 93 °C. In the field experiment, the phyllochron among genotypes varied from 48 to 60 °C with a mean phyllochron across genotypes of 53 °C. Based on the 95% confidence intervals for the phyllochron estimate, no difference among genotypes was detected in the field. By contrast in the pot experiment, genetic differences were found between low-phyllochron genotype ICG 4750 (81 °C) and four high-phyllochron genotypes ICGV 00350 (102 °C), ICG 3584 (102 °C), ICG 91114 (98 °C), and 55–437 (95 °C) (Table 1). However, the phyllochron values in the pots were far higher than those recorded earlier Leong and Ong (1983) and Craufurd et al. (1997), which were in line with those recorded here in the field.

### 3.2 Plant leaf area

Leaf area development showed genotypic differences in the both field and pot experiments. In the field (Table 2), PLA showed highly significant genotypic difference at the three harvest dates (16, 40, 70 DAS, which corresponded to cumulative temperature units 289, 688 and 1198 °C). At 289 °C, ICG 4750 had the greatest PLA (93 cm^2^ plant^−1^) among genotypes. Seven genotypes had PLA ranging from 67 to 80 cm^2^ plant^−1^, and the lowest PLA were obtained by Fleur 11 (47 cm^2^ plant^−1^) and 55–437 (53 cm^2^ plant^−1^). At 688 °C, genotype ICG 91114 showed the highest PLA (718 cm^2^ plant^−1^) whereas the remaining 9 genotypes all had PLA ranging between 330 and 492 cm^2^ plant^−1^. In reversal of the first date of observations, at 1198 °C-days Fleur 11 had the largest PLA (5709 cm^2^ plant^−1^) whereas the remaining genotypes had PLA in the range of 2435 to 3319 cm^2^ plant^−1^.

In the pot experiment (Table 3), large differences in PLA among genotypes were again observed on each measurement date. On the first date, JL24 had the greatest PLA. However, on nearly all succeeding dates ICGV 00350 had the greatest PLA eventually reaching 2837 cm^2^ plant^−1^ for the final measurement. ICG 12697 also consistently had high PLA with a final area of 2006 cm^2^ plant^−1^. After initially having a high PLA, ICG 3584 had the lowest PLA on the final two measurement dates.

Overall, the PLA of the plants grown in the pots was well below that of field-grown plants due to slow leaf appearance rate (high phyllochron) in the pots. At a young vegetative stage, the overall mean of PLA in pots was 39 cm^2^ plant^−1^ at 271 °C compared to 70 cm^2^ plant^−1^ in the field at 289 °C (Table 2, Table 3). Later on at 958 °C, plants grown in pots had a mean PLA of 425 cm^2^ plant^−1^, while this PLA was obtained in the field in only 688 °C. The mean PLA measured on the final harvest date in the field (temperature unit = 1198 °C) was 3150 cm^2^ plant^−1^ in contrast to a mean of only 1831 cm^2^ plant^−1^ obtained in the pots after 1681 °C.

### 3.3 Leaf area and main stem node number

**Fig. 2:**
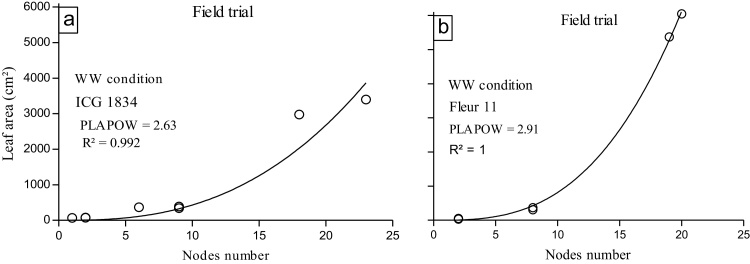
Illustration of plant leaf area considered as a function of main stem node number described by the power function for two peanut genotypes (a) ICG 1834 and (b) Fleur 11 in Exp. 2.

The relationship between leaf area and main stem node number was well described by the power function given in Eq. (2) as illustrated in Fig. 2. The results of the two genotypes presented in Fig. 2 represents the extreme values for the PLAPOW exponent obtained in the field in Exp 2. In examining results from both the field and pots, the R^2^ for all genotype was equal to 0.98 or greater (Table 4).

The values of PLAPOW obtained in the pot experiment ranged across genotypes between 2.83 (ICGV 00350) and 2.45 (ICG 4750) with a mean of 2.57. Fleur 11 (PLAPOW = 2.49) and ICG 4750 (PLAPOW = 2.45) recorded the lowest PLAPOW. These two genotypes were different from ICGV 00350 and ICG 1834, which had the highest PLAPOW values of 2.83 and 2.70, respectively.

**Fig. 3:**
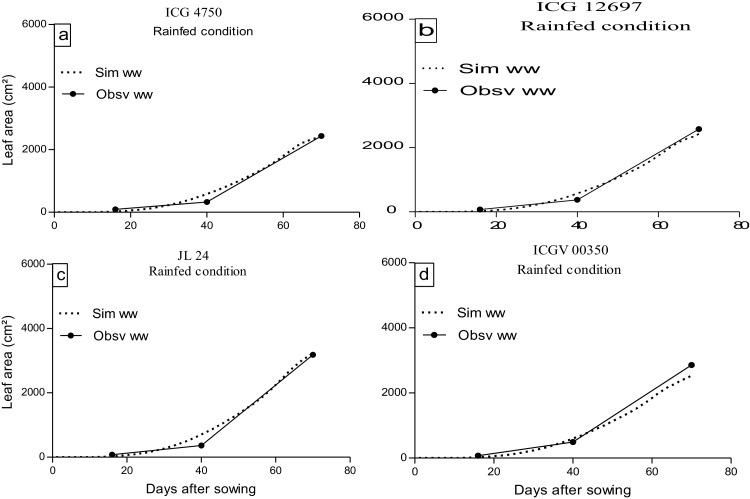
Observed and simulated leaf area evolution of four genotypes of peanut [ICG 4750 (a), ICG 12697 (b), JL 24 (c), ICGV 00350 (d)] growth in Field (Exp.2). Solid line represent observed leaf area and dashed line are simulated leaf area.

Field results for PLAPOW in Exp 2 (Table 4) gave values ranging from 2.63 for ICG 1834 to 2.91 for Fleur 11. Based on the fairly narrow confidence interval for the genotypes, PLAPOW was significantly different between the extreme genotypes. The value of PLAPOW for Fleur 11 was significantly different from 55 to 437 and JL24 with PLAPOW of 2.81 and 2.80, respectively. The lowest values of PLAPOW were estimated for genotypes ICG 1834 and ICG 3584 (Fig. 3).

The fact that the mean PLAPOW parameter was somewhat lower in pots (2.57) than in the field (2.74) indicates that the pot growth conditions likely also limited leaf expansion. However, the individual values of PLAPOW for each genotype were similar in the field and in pots for four genotypes (IGC 1834, ICG 3584, TMV2, and ICG 00350) out of ten (Table 4). For the six remaining genotypes their PLAPOW value obtained in the pot experiment were between 0.18 and 0.42 lower than obtained in the field experiment.

### 3.4 Assessing genetic variation for PLAPOW in a larger range of genotypes

In Exp 3, there was a narrow variation in PLAPOW across genotypes, although differences between genotypes were significant (Table 5). In Exp 3, PLAPOW mean was 2.71 and ranged between 2.83 to 2.56. The genotypes ICG 11088 (2.56), ICGS 44 (2.60) and ICG 12697 (2.62) had the lowest PLAPOW. These three low PLAPOW genotypes were in the same confidence intervals as eleven genotypes (Table 5), but were less than six genotypes (ICG 4750, FLEUR 11, 55–437, ICGV 02266, ICG 15287, and ICGV 01232) in which PLAPOW ranged between 2.74 and 2.83. In addition to the three low PLAPOW genotypes, three additional low PLAPOW genotypes (ICGV 02189 (2.66), JL 24(2.66), ICG 8751 (2.68)) were also different from the six highest PLAPOW genotypes. In the ten genotypes similar in the two experiments (Exp2 and 3), FLEUR 11, ICGV 00350, ICG 4750, ICGV 91114 and 55–437 had the highest PLAPOW in the two experiments (Exp2 and 3), while genotypes ICG1834, ICG 12697 and TMV2 had the lowest PLAOW (Table 4, Table 5). Genotypes ICGV 00350, ICG 4750, ICGV 91114, and TMV2 had stable PLAPOW among the two experiments, but JL-24, FLEUR 11, ICG 12697and 55–437 PLAPOW values decrease in Exp 3. The fact that genotypes like ICG1834 did not rank the same across both experiments indicates there was some degree of genotype-by-environment (G × E) interaction for the PLAPOW coefficient.

### 3.5 Assessing groundnut subspecies and density effects on PLAPOW

In Exp 4, there was also genetic variation across the four densities. In densities 10, 20 and 40 plant m^−2^, genotype ICG 13723 had a lower PLAPOW (2.57, 2.55 and 2.57 respectively) than the three others genotypes (Table 6). In density 30 plant m^−2^ also, genotypes ICG 13723 showed lowest PLAPOW (2.65), although it was different from Fleur 11 (2.77) only (Table 6). In this experiment, there was no significant difference in the PLAPOW between botanical groups except at the 30 plant m^−2^ density. The slightly lower PLAPOW of Virginia types compared to Spanish types was mostly explained by the lower PLAPOW of ICG13723. In this experiment where Virginia ICG13723 was replaced by ICG4598, there was no genetic variation of PLAPOW. Similarly there was no PLAPOW difference between botanical types, and the PLAPOW in Virginia and Spanish types was 2.64 and 2.65 respectively in density 20; 2.7 and 2.73 in density 30; 2.74 and 2.77 in density 40 (Table 6).

Sowing density did not show major effects on the PLAPOW coefficient (Table 6). In Exp. 4 (Niger), the average PLAPOW didn’t vary with density for both Virginia and Spanish types. A slight contrast to this was Exp5 in India, where slight increases in the PLAPOW occurred as the density increased.

Another set of analyses was also done to consider the possibility of a plant type and density effect across both environments. While we were able to detect genotype × density interaction effects for the PLAPOW parameter, the differences in leaf area prediction between the more complex (that incorporated genotype × density effects) and simpler models was negligible in the context of simulating the crop using SSM (data not shown). Therefore, density was not considered in the model for simulating groundnut canopy development in the scope of SSM.

### 3.6 Robustness of the model

Simulation of the leaf area were done at both locations, using simulations of a standard cultivar, using a common phyllochron value of 53 °C and a common PLAPOW parameter value of 2.71, which was the average of the values generated above. Leaf area simulations were compared with observed leaf area in the field experiments (Exp 2, 4, and 5). The phyllochron (53 °C) recorded in field (Exp.2) were used to simulate all botanical types. The mean PLAPOW value across Exp2 and Exp3 for the 10 genotypes that were common to both experiments was used to simulate the leaf area development of these 10 genotypes, as measured in Exp.2, using a density of 20 plant m^−2^. In Exp 4 and 5, A single PLAPOW 2.71 was used to simulate leaf area of all botanical types (Spanish and Virginia). In this case, simulations were done using the densities that were used in either sites, Niger or India.

**Fig. 4:**
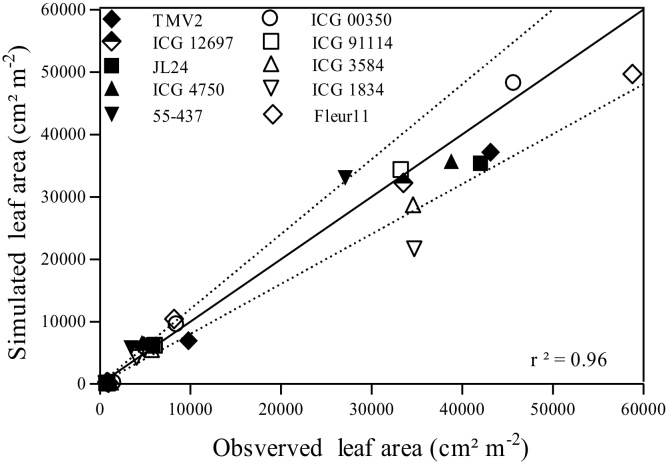
Simulated versus measured leaf area of ten peanut genotypes (Spanish type) from Exp.2, using the PLAPOW value for each genotype (mean of PLAPOW value from Exp2 and Exp3). The 20 % ranges of discrepancy between simulated and measured are indicated by dashed lines. Solid line is 1:1 line.

The simulation results matched well with experimental results, the model results for leaf area was very close to observed values in experiment 2 (Fig. 4). Most of data points were in the range of ±20% discrepancy with an overall very strong and significant relationship (R^2^ = 0.96, P < 0.0001). The coefficients of determination (R^2^) in the 10 genotypes in Exp 2 were very high and ranged between 0.97 and 0.99.

**Fig. 5:**
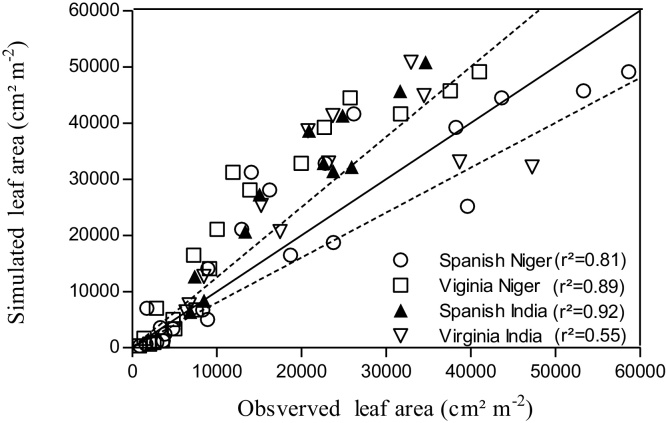
Simulated versus measured leaf area in peanut genotypes (Spanish and Virginia types) in Exp.3,4 and 5, using a single phyllochron of 53ºC and a single PLAPOW value of 2.71. The 20% ranges of discrepancy between simulated and measured are indicated by dashed lines. Solid line is 1:1 line. n = number of observations.

In the density experiments, results showed significant linear correlations between simulated and observed leaf area in Spanish (R^2^ = 0.81, P < 0.0001) and Virginia types (R^2^ = 0.89, P < 0.0001) in Niger (Fig. 5), and in Spanish (R^2^ = 0.92, P < 0.0001) and Virginia types (R^2^ = 0.55, P = 0.004) in India (Fig. 5). The coefficients of determination between observed and simulated leaf area in different densities and botanical types (Spanish and Virginia) was very high and significant (density 10 plant m^−2^, R^2^ = 0.64 and 0.99; density 20 plant m^−2^, R^2^ = 0.99 and 0.97; density 30 plant m^−2^, R^2^ = 0.91 and 0.91; density 40 plant m^−2^, R^2^ = 0.87 and 0.89 Spanish and Virginia respectively) in Niger experiments. In India also, coefficient of determination between simulated and observed leaf area were very strong, in density 25 plant m^−2^ (R^2^ = 0.96 and 0.92 Spanish and Virginia respectively), density 33 plant m^−2^ (R^2^ = 0.91 and 0.76 Spanish and Virginia respectively) and density 50 plant m^−2^ (R^2^ = 0.87 and 0.34 Spanish and Virginia respectively) (data not represented). Overall, there was a slight over-prediction of the leaf areas in the Virginia type in Niger, and of the Spanish types in India, which could be simply due to experimental conditions limiting the leaf area development (biotic stress for instance).

## 4 Discussion

It has been documented for peanut (Leong and Ong, 1983) and many crops, soybean (Sinclair, 1984), cowpea (Craufurd et al., 1998), chickpea (Soltani et al., 2006), pigeonpea (Ranganathan et al., 2001) that leaf area development is closely associated with cumulative daily temperature units. The observations of phyllochron in this study confirmed the importance of temperature in determining plant development. The mean field phyllochron of 53 °C obtained in the field experiment for the ten studied genotypes matches well with the phyllochron reported by Leong and Ong (1983) for peanut of 56 °C. The objective of this experiment was to determine if genetic diversity existed for leaf development parameters in the simplified model describing PLA development among ten lines selected from the ICRISAT reference collection. Under field conditions (Exp 2) no significant difference in phyllochron was found among these genotypes with values within the narrow range of 48 to 60 °C. This result is consistent with the failure to identify genetic variability among maize germplasm (van Esbroeck et al., 2008).

An unexpected outcome of this study was the large difference in phyllochron obtained between the field and pot experiments. This was unexpected as van Esbroeck et al. (2008) found in maize similar phyllochron for field and pot experiments. The phyllochron observed for field-grown peanut plants was nearly half of that observed in pots. One possibility to explain the higher phyllochron in the pot study as compared to the field studies may be a result of the relatively small volume of pots (6 L) used in this study. Based on a meta-analysis of 65 studies, Poorter et al. (2012) concluded that no more than 1 g plant mass per L of pot volume should exist in pot studies to avoid growth limitations. In our pot study, plant mass averaged 37 g plant^−1^ by the 9th harvest (1681 °C), which means the pot volume was well below the criterion of Poorter et al. (2012). Another potential explanation was the fact that this experiment was carried out over the winter season and temperature conditions for the rhizosphere of the pots could have been colder than those in the field, but the temperature data for the pot data were also those from the weather station.

Genetic differences in leaf area developments as defined by the PLAPOW coefficient (Eq. (2)) were found in both Exp 2 and Exp 3 field studies, where 10 and 20 Spanish genotypes were tested. There were some differences in the ranking of the PLAPOW between these two experiments, indicating G × E interactions (data not shown). Exp 2 was carried out during the summer season in Niger, characterized by high evaporative demand, whereas Experiment 3 was carried out toward the end of the rainy season, with much lower evaporative demand. There is evidence in maize that vapor pressure deficit affects the leaf expansion process (Reymond et al., 2003). Our interpretation is that vapor pressure deficit could have affected the leaf expansion processes in a different way across genotypes, leading to the GxE interactions in the PLAPOW values. More work would be needed to assess the possibility of having such interactions. The variation in Exp 4, was between one Virginia genotypes (ICG 13723) and three others genotypes (ICG 2777 (Virginia type) and Fleur 11, ICG 1834 (Spanish type)). All these variations occurred in Niger experiments, not in India, where the Virginia genotype having the lowest PLAPOW was not used. This difference of leaf area development between the two localities could also result from an environmental effect.

The variation found in Exp 4 among densities was narrow with only a slight difference between lower and higher densities in India but none in Niger. Therefore, it was concluded that in this groundnut crop model there need not be an addition of a density factor controlling the PLAPOW, in contradiction with results in chickpea (Soltani and Sinclair, 2011). Difference in the PLAPOW between the two botanic groups (Virginia and Spanish) was also small (only at one density in Niger and none in India), and especially accounted for one genotype (ICG13723). A larger set of Virginia genotypes would need to be tested to confirm these slight PLAPOW differences between botanical types. The difference of responses in the two environments (Sadoré/Niger and Patancheru/India) could be the difference of soil which can influence leaf expansion as a result of difference of roots development. In Sadoré, the soil is 90% sandy soil (West et al., 1984), easy for root development, while Patancheru soil is red soil (Alfisol) more compact up to 55% of clay (Bhattacharyya et al., 2007). Sandy soil could alleviate effect of density compare to clay soil where root penetration in soil profile could be difficult.

Therefore, genetic variation in leaf area development was identified in this study that was related to the extent of leaf area development associated with increasing main stem node. An attempt to increase early season leaf area development might be achieved by using the lines with high PLAPOW. These results indicate that 55–437 might be an attractive candidate parental line to achieve high leaf area since it expressed high PLAPOW values under both field and pot experiments. Peanut leaf area prediction using the simple model used here for describing leaf area development showed good correlation between simulated and observed values of leaf area, across the diverse range of growth and environmental conditions for different genotypes. These results matched with previous study (Soltani and Sinclair, 2011) in chickpea with SSM-chickpea.

## 5 Conclusion

There was no genetic variation in the phyllochron, which based on the data collected in these experiments can be fixed at 53 °C. There were genotypic differences in the PLAPOW, although there seemed to be also an effect of the environment, most likely related to vapor pressure deficit on PLAPOW. There was no botanical-type effect on the PLAPOW, at least with the narrow range of Virginia genotypes that were used. Similarly, density had virtually no effect on the PLAPOW. Therefore, there is need for further investigation of a possible genotype-by-environment effect on the PLAPOW and until exploration of a larger range of Spanish and Virginia genotypes, a PLAPOW of 2.71 can be safely used for modelling leaf area development in groundnut.

## Figures and Tables

**Table 1:** Phyllochron (°C) of ten genotypes of peanut measured in field (Exp. 2) and pot (Exp. 1) experiments.

Genotypes	Field					POT				
	phyll	SE	CI 95%	R^2^	RMSE	phyll	SE	CI 95%	R^2^	RMSE
55–437	**60**	2.4	55–66	0.99	0.7	**95**	4.1	87–103	0.93	1.0
ICGV 00350	**56**	4.6	45–67	0.96	1.6	**102**	6.9	88–116	0.86	1.4
ICG 12697	**55**	4.1	46–65	0.96	1.5	**91**	3.4	84–98	0.95	0.9
FLEUR 11	**52**	2.7	46–58	0.98	1.1	**85**	3.1	78–91	0.95	0.9
ICG 4750	**56**	4.3	46–66	0.96	1.5	**81**	2.6	76–86	0.96	0.9
TMV2	**50**	2.8	43–57	0.98	1.3	**92**	4.3	83–101	0.92	1.1
JL24	**52**	5.0	40–64	0.94	2.1	**91**	2.3	86–96	0.98	0.6
ICGV 91114	**52**	2.6	46–58	0.98	1.1	**98**	3.9	90–106	0.94	0.9
ICG 3584	**53**	4.3	43–63	0.96	1.7	**102**	3.5	95–109	0.96	0.7
ICG 1834	**48**	4.0	39–58	0.95	1.9	**97**	5.1	86–107	0.90	1.2
**All Geno**	**53**	**1.3**	**51–56**	**0.95**	**1.6**	**93**	**1.5**	**90–96**	**0.91**	**1.2**

Phyll = Phyllochron (°C), SE = standard error, 95% CI = 95% Confidence Intervals, SSE = Error sum of squares, SSG = Corrected total sum of squares, MSE = Error mean square.

**Table 2:** Leaf area (cm^2^) measured in field experiment (Exp. 2) on three dates after sowing (cumulated degres after sowing, °C) listed by the cumulative temperature on each of the dates.

Entry Name	289 °C	688 °C	1198 °C
55–437	53.1c	341.3b	2451b
ICGV 00350	72.7b	491.9b	2856b
ICG 12697	73.6b	370.6b	2577b
FLEUR 11	45.9c	345.7b	5709a
ICG 4750	92.8a	329.7b	2435b
TMV2	68.5b	454.8b	3155b
JL24	79.7b	361.2b	3183b
ICGV 91114	75.0b	707.7a	3319b
ICG 3584	66.7b	440.9b	2659b
ICG 1834	73.1b	368.4b	3154b
Mean	**70**	**421**	**3150**
L.s.d	19.109	110.750	1115.9
F prob.	0.005	<0.001	0.001

L.s.d = least significant difference; F prob = Probability at 5% level. Values identified with the same letter are not statistically different from each other based on Bonferroni test at a significance level of 0.05.

**Table 3:** Leaf area (cm^2^) measured in pot experiment (Exp. 1) on nine dates listed by the cumulative temperature on each of the dates after sowing (cumulated °C after sowing).

Entry Name	Cumulated °C after sowing								
	271	432	578	711	837	958	1069	1430	1681
55–437	25.74g	75.20b	104.1g	280.8c	393.7d	714.4a	687.7b	800d	1861e
ICGV 00350	49.26b	122.29a	153.1c	361.8a	567.4a	696.9a	765.2a	844d	2837a
ICG 12697	39.70d	74.96b	145.2d	294.7b	459.7b	332.8e	566.2c	1706a	2006d
FLEUR 11	44.92c	129.08a	158.1c	214.3d	456.5b	395.1d	649.5b	1774a	1841e
ICG 4750	31.94f	88.66c	144.9d	196.8e	277.6f	274.0f	656.1b	1182c	2705b
TMV2	42.65c	73.60b	107.4f	331.8b	467.0b	342.5e	693.7b	1033c	1420f
JL24	58.44a	88.17c	180.6b	195.3e	379.3e	285.5f	804.3a	1075c	1467f
ICGV 91114	23.95h	83.65c	152.8c	129.7f	162.3h	307.8e	430.2d	1266b	2268c
ICG 3584	41.71c	104.41b	197.6a	155.6e	264.5g	416.5c	636.7b	596e	766g
ICG 1834	34.53e	89.71c	140.8e	186.0e	399.3c	485.0b	551.2c	1249b	1136g
Mean	**39**	**93**	**148**	**235**	**383**	**425**	**644**	**1153**	**1831**
L.s.d	**5.32**	**14.66**	**20.16**	**46.05**	**67.92**	**69.52**	**107.0**	**215.8**	**395.5**
F prob.	**<0.001**	**<0.001**	**<0.001**	**<0.001**	**<0.001**	**<0.001**	**<0.001**	**<0.001**	**<0.001**

L.s.d = least significant difference; F prob = Probability at 5% level. Values identified with the same letter are not statistically different from each other based on Bonferroni test at a significance level of 0.05.

**Table 4:** Comparison of the exponential coefficient PLAPOW (coefficient defining relationship between plant leaf area and node number on the main stem) of ten genotypes of peanut derived from field (Exp.2) and pot (Exp.1) observations.

Genotype	Field					Pots				
	PLAPOW	se	95% CI	R^2^	RMSE	PLAPOW	se	95% CI	R^2^	RMSE
55–437	**2.81**	0.014	2.78–2.84	0.999	130	**2.63**	0.022	2.58–2.67	0.997	119
ICGV 00350	**2.73**	0.049	2.61–2.84	0.983	665	**2.83**	0.037	2.76–2.91	0.994	199
ICG 12697	**2.71**	0.030	2.64–2.78	0.993	386	**2.51**	0.025	2.46–2.56	0.997	113
FLEUR 11	**2.91**	0.003	2.9–2.92	1.000	78	**2.49**	0.027	2.43–2.54	0.996	128
ICG 4750	**2.72**	0.025	2.66–2.78	0.995	300	**2.45**	0.021	2.4–2.49	0.997	91
TMV2	**2.71**	0.016	2.67–2.75	0.998	208	**2.61**	0.030	2.55–2.67	0.996	131
JL24	**2.8**	0.044	2.7–2.91	0.985	553	**2.61**	0.025	2.56–2.66	0.997	117
ICGV 91114	**2.76**	0.031	2.69–2.84	0.992	553	**2.52**	0.028	2.46–2.57	0.996	65
ICG 3584	**2.67**	0.039	2.58–2.76	0.988	511	**2.65**	0.023	2.61–2.7	0.998	90
ICG 1834	**2.63**	0.031	2.56–2.71	0.992	414	**2.70**	0.025	2.65–2.75	0.997	97
All genotypes	**2.74**	0.013	2.71–2.77	0.999	602	**2.57**	0.011	2.55–2.59	1.000	40

SE = standard error; 95% CI = 95% Confidence Intervals; RMSE = Root Mean Square Error.

**Table 5:** exponential coefficient PLAPOW (coefficient defining relationship between plant leaf area and node number on the main stem) of twenty genotypes of peanut derived from field observations (Exp. 3).

Genotypes	PLAPOW	se	95% CI	R^2^	RMSE
55–437	**2.75**	0.014	2.72–2.79	0.96	161
ICGV 00350	**2.74**	0.039	2.65–2.83	0.72	264
ICG 12697	**2.62**	0.032	2.54–2.69	0.78	281
ICG 4750	**2.74**	0.012	2.71–2.76	0.97	149
TMV2	**2.69**	0.007	2.68–2.71	0.99	88
JL 24	**2.66**	0.032	2.58–2.73	0.79	326
ICGV 91114	**2.75**	0.030	2.68–2.82	0.83	446
ICG 3584	**2.77**	0.034	2.69–2.85	0.81	442
ICG1834	**2.70**	0.037	2.61–2.78	0.74	372
FLEUR 11	**2.76**	0.019	2.71–2.80	0.95	212
ICG 97183	**2.72**	0.031	2.65–2.79	0.83	370
ICGV 97182	**2.75**	0.049	2.64–2.86	0.69	563
ICGV 02266	**2.82**	0.033	2.74–2.90	0,83	374
ICGV 02189	**2.66**	0.040	2.57–2.76	0.66	316
ICG11088	**2.56**	0.045	2.46–2.67	0.59	488
ICG 8751	**2.68**	0.033	2.60–2.75	0.78	324
ICGV 01232	**2.82**	0.025	2.76–2.87	0.89	328
ICGS 44	**2.60**	0.019	2.55–2.64	0.93	172
ICG 15287	**2.83**	0.039	2.74–2.92	0.79	466
ICGV 99001	**2.77**	0.054	2.64–2.91	0.68	395
All genotypes	**2.71**	**0.007**	**2.70–2.72**	**0.76**	**397**

SE = standard error; 95% CI = 95% Confidence Intervals; RMSE = Root Mean Square Error.

**Table 6:** variation of exponential coefficient PLAPOW (coefficient defining relationship between plant leaf area and node number on the main stem) of 4 genotypes of peanut in different sowing density from field observations (Exp. 4 and 5). (Pla = PLAPOW, CI 95% = 95% Confidence Intervals).

Genotypes	D10			D20			D30			D40		
	Pla	CI 95%	R^2^	Pla	CI 95%	R^2^	Pla	CI 95%	R^2^	Pla	CI 95%	R^2^
Exp.4												
ICG 13723	**2.57**	2.50–2.64	0.79	**2.55**	2.51–2.60	0.88	**2.65**	2.60–2.70	0,9	**2.57**	2.53–2.61	0.88
ICG 2777	**2.77**	2.72–2.82	0.88	**2.75**	2.69–2.80	0.83	**2.75**	2.69–2.80	0,8	**2.68**	2.62–2.74	0.80
FLEUR 11	**2.71**	2.66–2.76	0.87	**2.77**	2.72–2.83	0.83	**2.77**	2.72–2.82	0,8	**2.74**	2.67–2.81	0.73
ICG 1834	**2.78**	2.71–2.84	0.76	**2.66**	2.60–2.72	0.72	**2.71**	2.65–2.78	0,8	**2.67**	2.62–2.72	0.87
All genotypes	**2.72**	2.69–2.75	0.77	**2.70**	2.69–2.75	0.77	**2.71**	2.68–2.74	0.79	**2.68**	2.65–2.70	0.78
Spanish type (mean)	**2.76**	2.72–2.80	0.80	**2.72**	2.72–2.80	0.80	**2.74**	2.70–2.78	0.80	**2.70**	2.66–2.74	0.78
Virginia type (mean)	**2.68**	2.63–2.73	0.76	**2.67**	2.63–2.73	0.76	**2.67**	2.63–2.70	0.81	**2.63**	2.59–2.67	0.78

Exp.5					D25			D33			D50	
ICG 4598				**2.64**	2.61–2.68	0.90	**2.71**	2.67–2.74	0.91	**2.75**	2.68–2.81	0.75
ICG 2777				**2.63**	2.58–2.68	0.75	**2.70**	2.65–2.75	0.86	**2.73**	2.66–2.79	0.77
Fleur 11				**2.63**	2.56–2.69	0.59	**2.74**	2.68–2.80	0.71	**2.75**	2.71–2.79	0.89
ICG 1834				**2.67**	2.59–2.75	0.49	**2.71**	2.67–2.75	0.84	**2.78**	2.71–2.85	0.60
All genotypes				**2.64**	2.62–2.67	0.73	**2.71**	2.69–2.73	0.85	**2.75**	2.72–2.77	0.76
Spanish type (mean)				**2.65**	2.60–2.69	0.52	**2.73**	2.69–2.76	0.77	**2.77**	2.73–2.80	0.76
Virginia type (mean)				**2.64**	2.61–2.67	0.84	**2.70**	2.67–2.73	0.88	**2.74**	2.69–2.78	0.76
